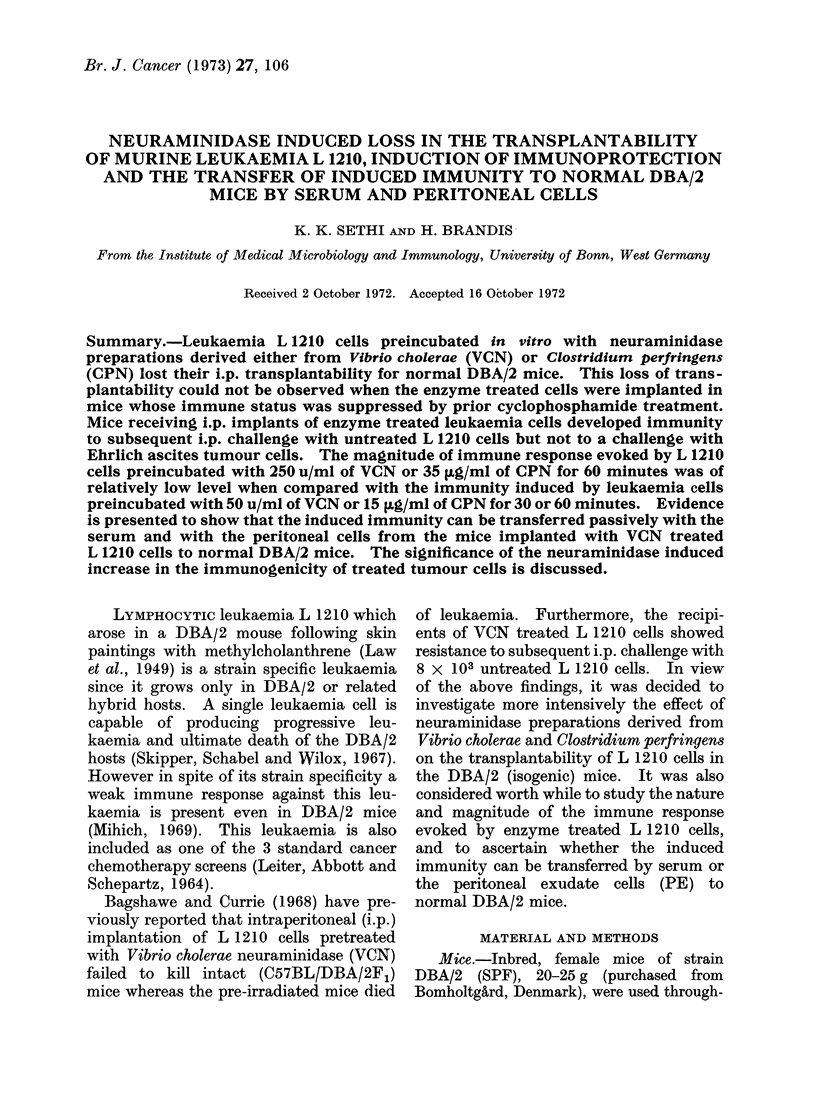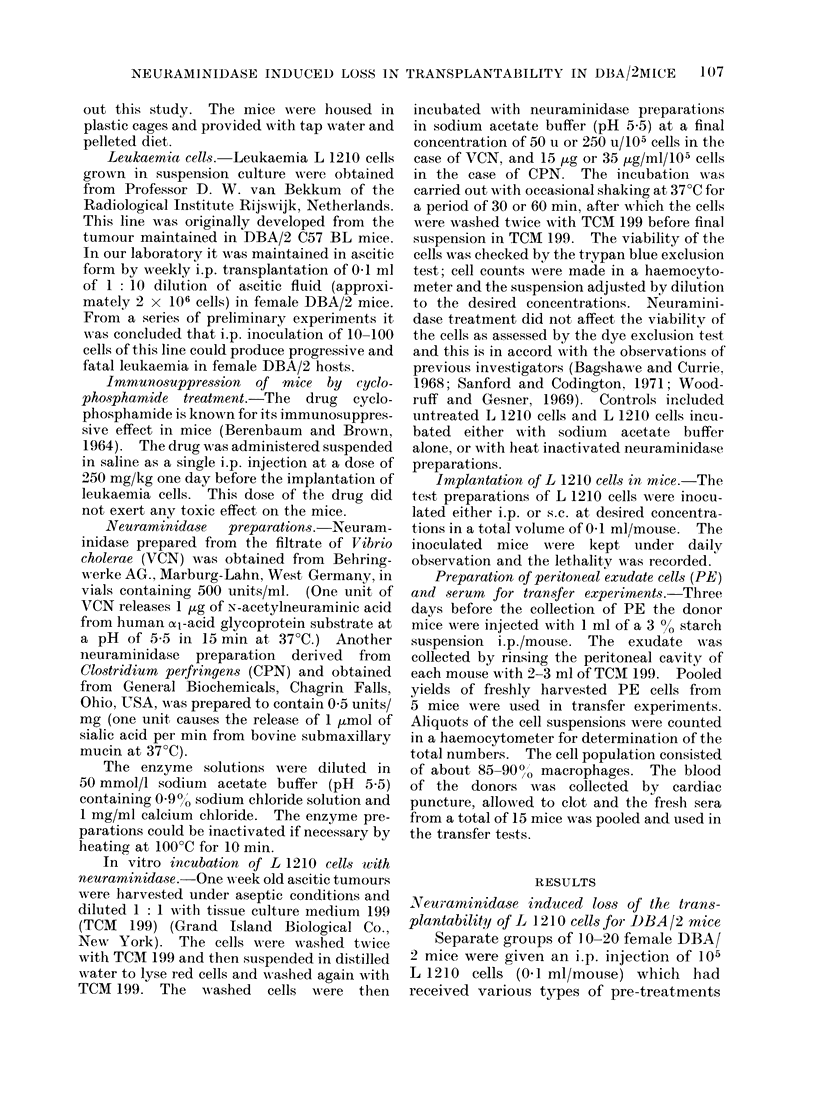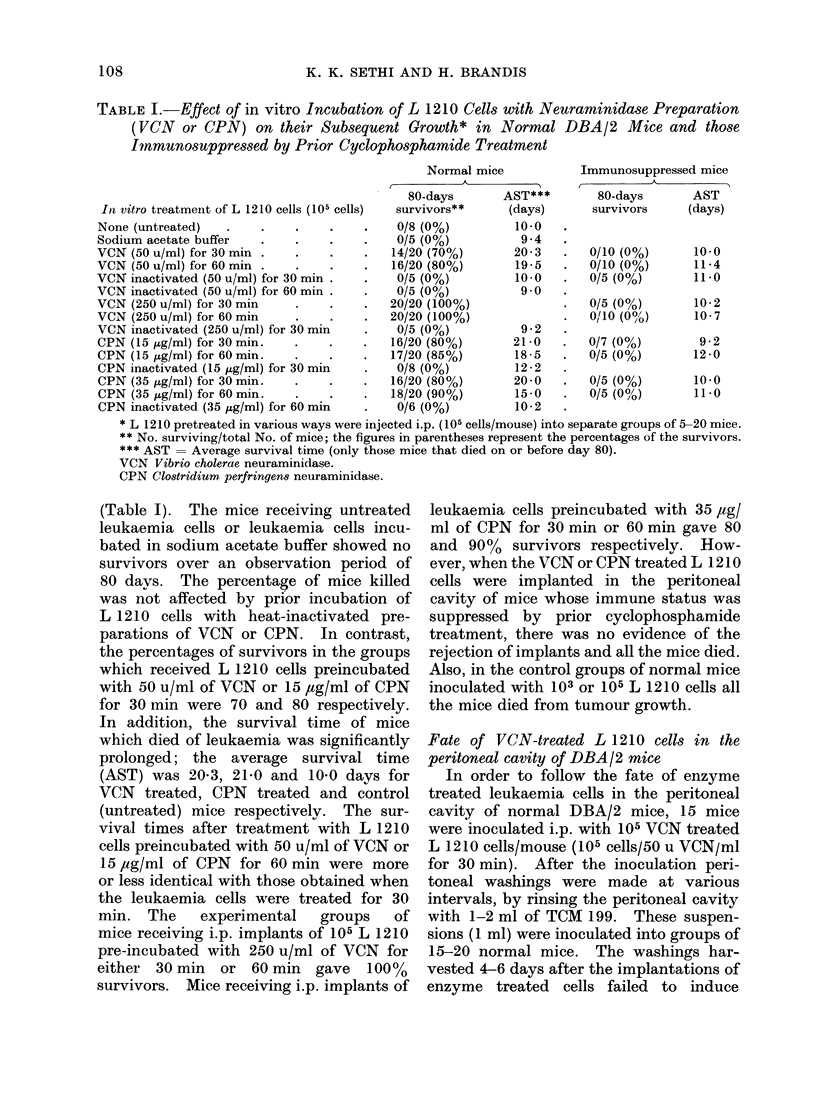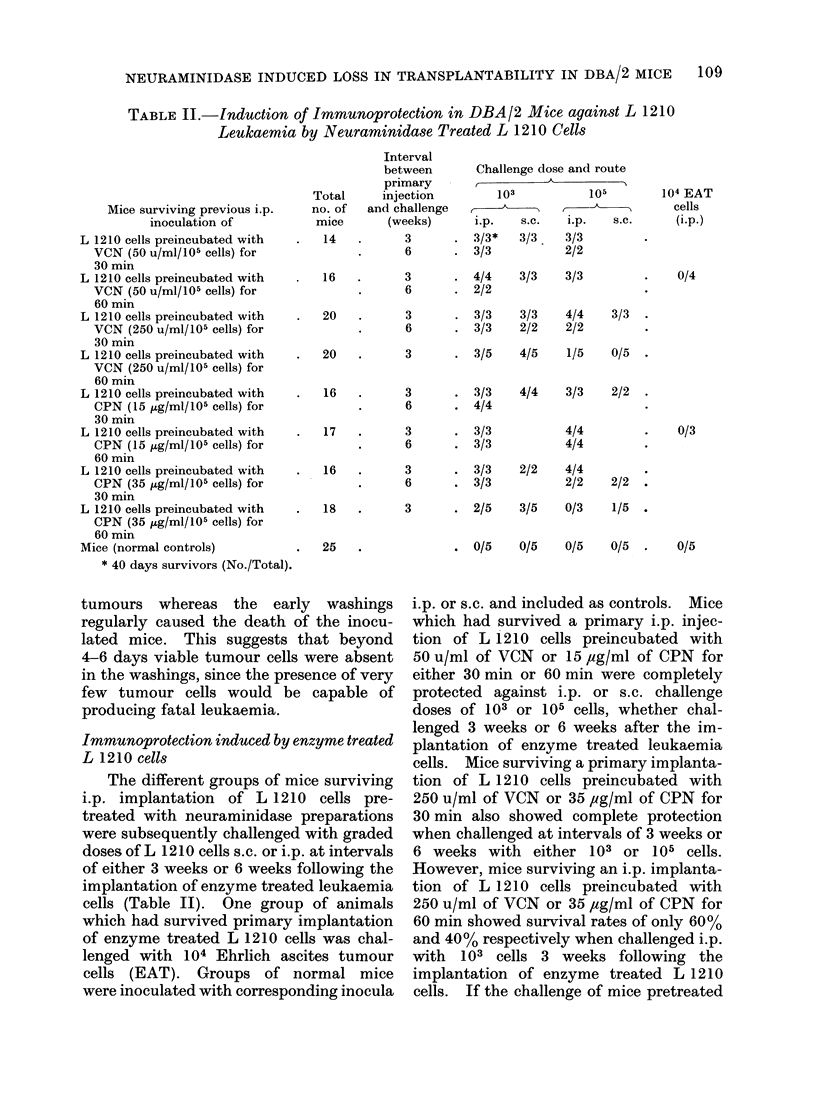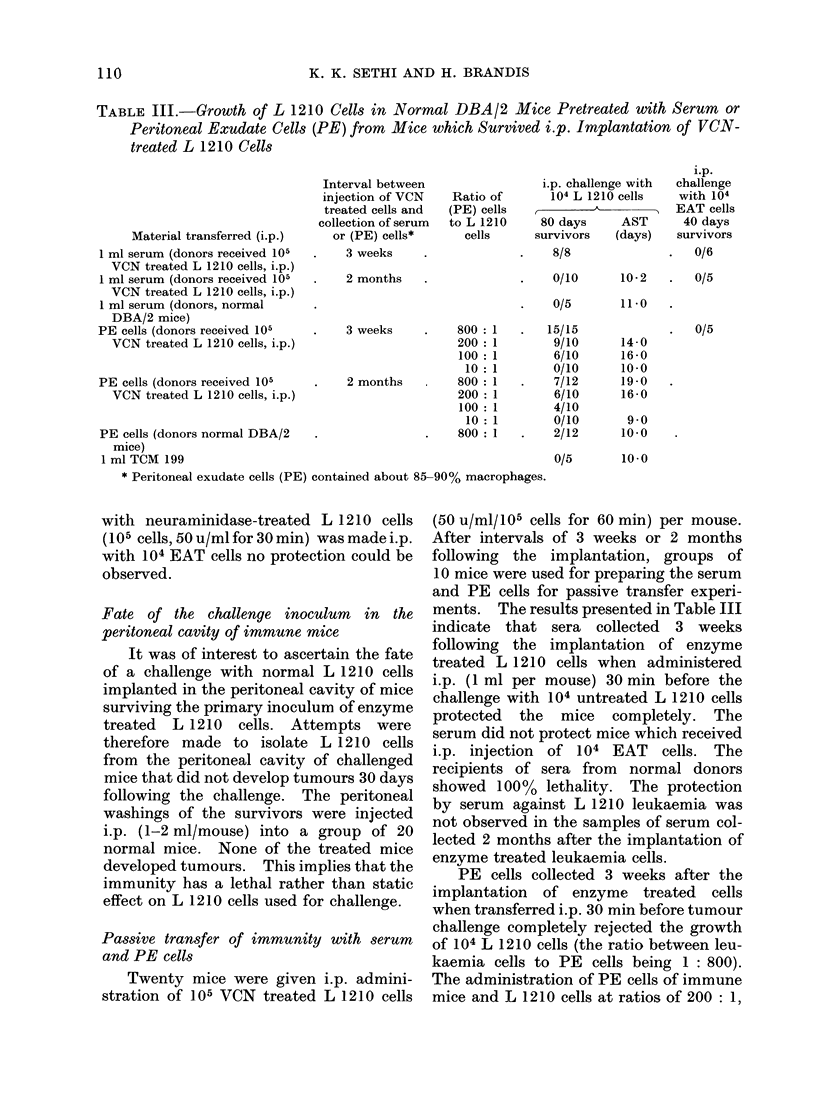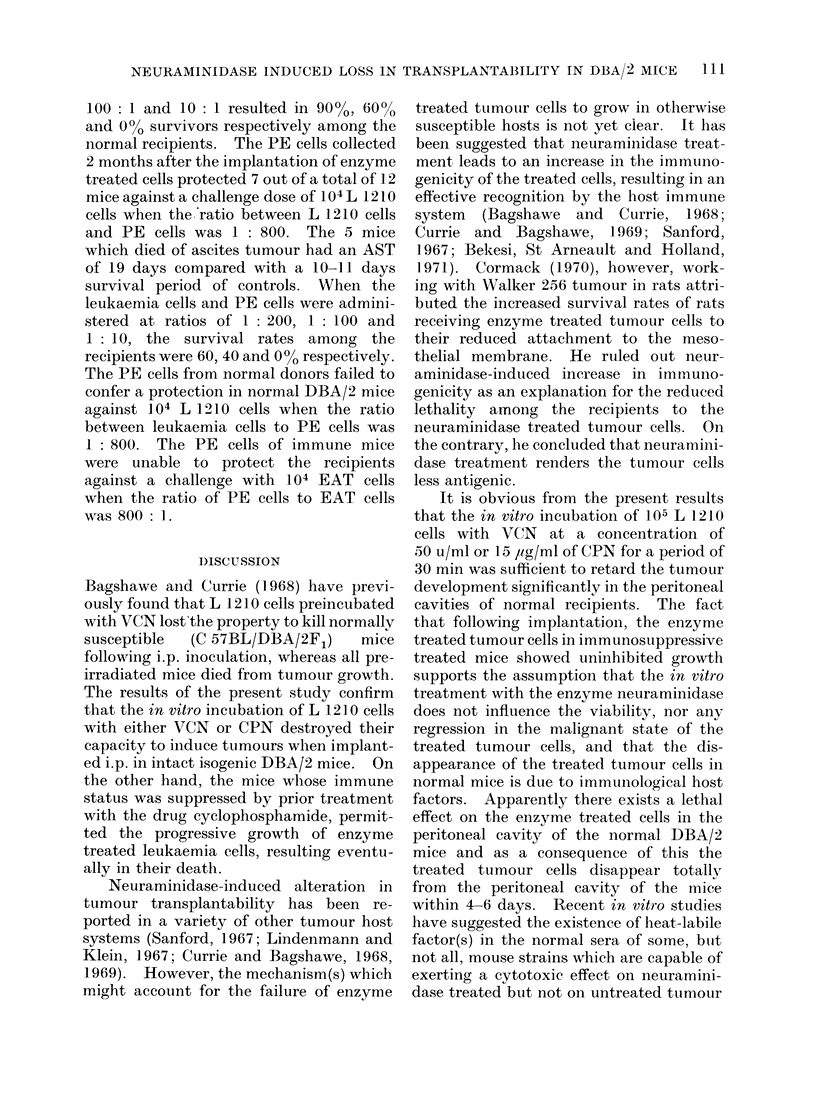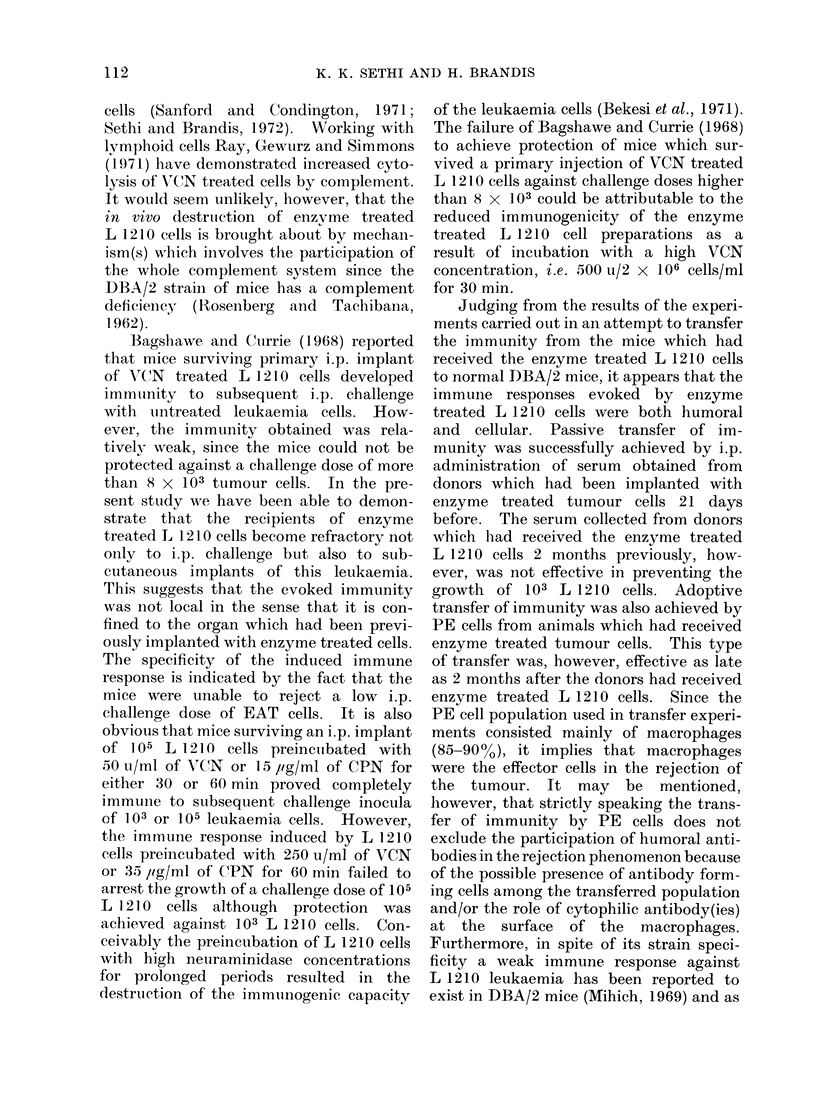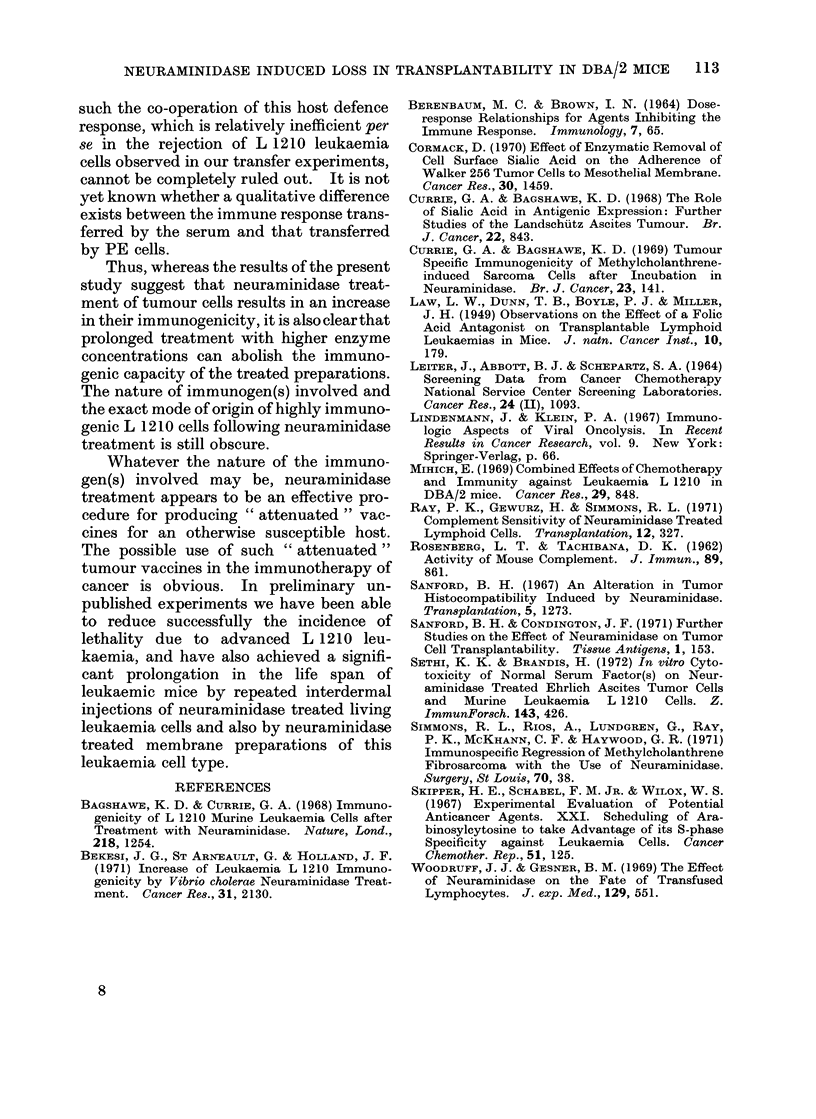# Neuraminidase Induced Loss in the Transplantability of Murine Leukaemia L 1210, Induction of Immunoprotection and the Transfer of Induced Immunity to Normal DBA/2 Mice by Serum and Peritoneal Cells

**DOI:** 10.1038/bjc.1973.14

**Published:** 1973-02

**Authors:** K. K. Sethi, H. Brandis

## Abstract

Leukaemia L 1210 cells preincubated *in vitro* with neuraminidase preparations derived either from *Vibrio cholerae* (VCN) or *Clostridium perfringens* (CPN) lost their i.p. transplantability for normal DBA/2 mice. This loss of transplantability could not be observed when the enzyme treated cells were implanted in mice whose immune status was suppressed by prior cyclophosphamide treatment. Mice receiving i.p. implants of enzyme treated leukaemia cells developed immunity to subsequent i.p. challenge with untreated L 1210 cells but not to a challenge with Ehrlich ascites tumour cells. The magnitude of immune response evoked by L 1210 cells preincubated with 250 u/ml of VCN or 35 μg/ml of CPN for 60 minutes was of relatively low level when compared with the immunity induced by leukaemia cells preincubated with 50 u/ml of VCN or 15 μg/ml of CPN for 30 or 60 minutes. Evidence is presented to show that the induced immunity can be transferred passively with the serum and with the peritoneal cells from the mice implanted with VCN treated L 1210 cells to normal DBA/2 mice. The significance of the neuraminidase induced increase in the immunogenicity of treated tumour cells is discussed.


*Br. J. Cancer* (1973) **27**, 106

### NEURAMINIDASE INDUCED LOSS IN THE TRANSPLANTABILITY OF MURINE LEUKAEMIA L 1210, INDUCTION OF IMMUNOPROTECTION AND THE TRANSFER OF INDUCED IMMUNITY TO NORMAL DBA/2 MICE BY SERUM AND PERITONEAL CELLS

K. K. SETHI AND H. BRANDIS

*From the Institute of Medical Microbiology and Immunology, University of Bonn, West Germany*

Received 2 October 1972. Accepted 16 October 1972

**Summary.—Leukaemia L 1210 cells preincubated *in vitro* with neuraminidase preparations derived either from *Vibrio cholerae* (VCN) or *Clostridium perfringens* (CPN) lost their i.p. transplantability for normal DBA/2 mice. This loss of transplantability could not be observed when the enzyme treated cells were implanted in mice whose immune status was suppressed by prior cyclophosphamide treatment. Mice receiving i.p. implants of enzyme treated leukaemia cells developed immunity to subsequent i.p. challenge with untreated L 1210 cells but not to a challenge with Ehrlich ascites tumour cells. The magnitude of immune response evoked by L 1210 cells preincubated with 250 u/ml of VCN or 35 μg/ml of CPN for 60 minutes was of relatively low level when compared with the immunity induced by leukaemia cells preincubated with 50 u/ml of VCN or 15 μg/ml of CPN for 30 or 60 minutes. Evidence is presented to show that the induced immunity can be transferred passively with the serum and with the peritoneal cells from the mice implanted with VCN treated L 1210 cells to normal DBA/2 mice. The significance of the neuraminidase induced increase in the immunogenicity of treated tumour cells is discussed.**

LYMPHOCYTIC leukaemia L 1210 which arose in a DBA/2 mouse following skin paintings with methylcholanthrene (Law *et al.*, 1949) is a strain specific leukaemia since it grows only in DBA/2 or related hybrid hosts. A single leukaemia cell is capable of producing progressive leukaemia and ultimate death of the DBA/2 hosts (Skipper, Schabel and Wilox, 1967). However in spite of its strain specificity a weak immune response against this leukaemia is present even in DBA/2 mice (Mihich, 1969). This leukaemia is also included as one of the 3 standard cancer chemotherapy screens (Leiter, Abbott and Schepartz, 1964).

Bagshawe and Currie (1968) have previously reported that intraperitoneal (i.p.) implantation of L 1210 cells pretreated with *Vibrio cholerae* neuraminidase (VCN) failed to kill intact (C57BL/DBA/$2F_1$) mice whereas the pre-irradiated mice died of leukaemia. Furthermore, the recipients of VCN treated L 1210 cells showed resistance to subsequent i.p. challenge with $8 \times 10^3$ untreated L 1210 cells. In view of the above findings, it was decided to investigate more intensively the effect of neuraminidase preparations derived from *Vibrio cholerae* and *Clostridium perfringens* on the transplantability of L 1210 cells in the DBA/2 (isogenic) mice. It was also considered worth while to study the nature and magnitude of the immune response evoked by enzyme treated L 1210 cells, and to ascertain whether the induced immunity can be transferred by serum or the peritoneal exudate cells (PE) to normal DBA/2 mice.

### MATERIAL AND METHODS

*Mice.*—Inbred, female mice of strain DBA/2 (SPF), 20–25 g (purchased from Bomholtgård, Denmark), were used throughout this study. The mice were housed in plastic cages and provided with tap water and pelleted diet.

*Leukaemia cells.*—Leukaemia L 1210 cells grown in suspension culture were obtained from Professor D. W. van Bekkum of the Radiological Institute Rijswijk, Netherlands. This line was originally developed from the tumour maintained in DBA/2 C57 BL mice. In our laboratory it was maintained in ascitic form by weekly i.p. transplantation of 0·1 ml of 1 : 10 dilution of ascitic fluid (approximately $2 \times 10^6$ cells) in female DBA/2 mice. From a series of preliminary experiments it was concluded that i.p. inoculation of 10–100 cells of this line could produce progressive and fatal leukaemia in female DBA/2 hosts.

*Immunosuppression of mice by cyclophosphamide treatment.*—The drug cyclophosphamide is known for its immunosuppressive effect in mice (Berenbaum and Brown, 1964). The drug was administered suspended in saline as a single i.p. injection at a dose of 250 mg/kg one day before the implantation of leukaemia cells. This dose of the drug did not exert any toxic effect on the mice.

*Neuraminidase preparations.*—Neuraminidase prepared from the filtrate of *Vibrio cholerae* (VCN) was obtained from Behringwerke AG., Marburg-Lahn, West Germany, in vials containing 500 units/ml. (One unit of VCN releases 1 $\mu$g of N-acetylneuraminic acid from human $\alpha_1$-acid glycoprotein substrate at a pH of 5·5 in 15 min at 37°C.) Another neuraminidase preparation derived from *Clostridium perfringens* (CPN) and obtained from General Biochemicals, Chagrin Falls, Ohio, USA, was prepared to contain 0·5 units/mg (one unit causes the release of 1 $\mu$mol of sialic acid per min from bovine submaxillary mucin at 37°C).

The enzyme solutions were diluted in 50 mmol/l sodium acetate buffer (pH 5·5) containing 0·9% sodium chloride solution and 1 mg/ml calcium chloride. The enzyme preparations could be inactivated if necessary by heating at 100°C for 10 min.

In vitro *incubation of L 1210 cells with neuraminidase.*—One week old ascitic tumours were harvested under aseptic conditions and diluted 1 : 1 with tissue culture medium 199 (TCM 199) (Grand Island Biological Co., New York). The cells were washed twice with TCM 199 and then suspended in distilled water to lyse red cells and washed again with TCM 199. The washed cells were then incubated with neuraminidase preparations in sodium acetate buffer (pH 5·5) at a final concentration of 50 u or 250 u/$10^5$ cells in the case of VCN, and 15 $\mu$g or 35 $\mu$g/ml/$10^5$ cells in the case of CPN. The incubation was carried out with occasional shaking at 37°C for a period of 30 or 60 min, after which the cells were washed twice with TCM 199 before final suspension in TCM 199. The viability of the cells was checked by the trypan blue exclusion test; cell counts were made in a haemocytometer and the suspension adjusted by dilution to the desired concentrations. Neuraminidase treatment did not affect the viability of the cells as assessed by the dye exclusion test and this is in accord with the observations of previous investigators (Bagshawe and Currie, 1968; Sanford and Codington, 1971; Woodruff and Gesner, 1969). Controls included untreated L 1210 cells and L 1210 cells incubated either with sodium acetate buffer alone, or with heat inactivated neuraminidase preparations.

*Implantation of L 1210 cells in mice.*—The test preparations of L 1210 cells were inoculated either i.p. or s.c. at desired concentrations in a total volume of 0·1 ml/mouse. The inoculated mice were kept under daily observation and the lethality was recorded.

*Preparation of peritoneal exudate cells (PE) and serum for transfer experiments.*—Three days before the collection of PE the donor mice were injected with 1 ml of a 3 % starch suspension i.p./mouse. The exudate was collected by rinsing the peritoneal cavity of each mouse with 2–3 ml of TCM 199. Pooled yields of freshly harvested PE cells from 5 mice were used in transfer experiments. Aliquots of the cell suspensions were counted in a haemocytometer for determination of the total numbers. The cell population consisted of about 85–90% macrophages. The blood of the donors was collected by cardiac puncture, allowed to clot and the fresh sera from a total of 15 mice was pooled and used in the transfer tests.

### RESULTS

### *Neuraminidase induced loss of the transplantability of L 1210 cells for DBA/2 mice*

Separate groups of 10–20 female DBA/2 mice were given an i.p. injection of $10^5$ L 1210 cells (0·1 ml/mouse) which had received various types of pre-treatments

TABLE I.—*Effect of* in vitro *Incubation of L* 1210 *Cells with Neuraminidase Preparation (VCN or CPN) on their Subsequent Growth* in Normal DBA/2 Mice and those Immunosuppressed by Prior Cyclophosphamide Treatment*

| *In vitro* treatment of L 1210 cells ($10^5$ cells) | Normal mice 80-days survivors** | Normal mice AST*** (days) | Immunosuppressed mice 80-days survivors | Immunosuppressed mice AST (days) |
|---|---|---|---|---|
| None (untreated) | 0/8 (0%) | 10·0 | | |
| Sodium acetate buffer | 0/5 (0%) | 9·4 | | |
| VCN (50 u/ml) for 30 min | 14/20 (70%) | 20·3 | 0/10 (0%) | 10·0 |
| VCN (50 u/ml) for 60 min | 16/20 (80%) | 19·5 | 0/10 (0%) | 11·4 |
| VCN inactivated (50 u/ml) for 30 min | 0/5 (0%) | 10·0 | 0/5 (0%) | 11·0 |
| VCN inactivated (50 u/ml) for 60 min | 0/5 (0%) | 9·0 | | |
| VCN (250 u/ml) for 30 min | 20/20 (100%) | | 0/5 (0%) | 10·2 |
| VCN (250 u/ml) for 60 min | 20/20 (100%) | | 0/10 (0%) | 10·7 |
| VCN inactivated (250 u/ml) for 30 min | 0/5 (0%) | 9·2 | | |
| CPN (15 μg/ml) for 30 min. | 16/20 (80%) | 21·0 | 0/7 (0%) | 9·2 |
| CPN (15 μg/ml) for 60 min. | 17/20 (85%) | 18·5 | 0/5 (0%) | 12·0 |
| CPN inactivated (15 μg/ml) for 30 min | 0/8 (0%) | 12·2 | | |
| CPN (35 μg/ml) for 30 min. | 16/20 (80%) | 20·0 | 0/5 (0%) | 10·0 |
| CPN (35 μg/ml) for 60 min. | 18/20 (90%) | 15·0 | 0/5 (0%) | 11·0 |
| CPN inactivated (35 μg/ml) for 60 min | 0/6 (0%) | 10·2 | | |

* L 1210 pretreated in various ways were injected i.p. ($10^5$ cells/mouse) into separate groups of 5–20 mice.
** No. surviving/total No. of mice; the figures in parentheses represent the percentages of the survivors.
*** AST = Average survival time (only those mice that died on or before day 80).
VCN *Vibrio cholerae* neuraminidase.
CPN *Clostridium perfringens* neuraminidase.

(Table I). The mice receiving untreated leukaemia cells or leukaemia cells incubated in sodium acetate buffer showed no survivors over an observation period of 80 days. The percentage of mice killed was not affected by prior incubation of L 1210 cells with heat-inactivated preparations of VCN or CPN. In contrast, the percentages of survivors in the groups which received L 1210 cells preincubated with 50 u/ml of VCN or 15 μg/ml of CPN for 30 min were 70 and 80 respectively. In addition, the survival time of mice which died of leukaemia was significantly prolonged; the average survival time (AST) was 20·3, 21·0 and 10·0 days for VCN treated, CPN treated and control (untreated) mice respectively. The survival times after treatment with L 1210 cells preincubated with 50 u/ml of VCN or 15 μg/ml of CPN for 60 min were more or less identical with those obtained when the leukaemia cells were treated for 30 min. The experimental groups of mice receiving i.p. implants of $10^5$ L 1210 pre-incubated with 250 u/ml of VCN for either 30 min or 60 min gave 100% survivors. Mice receiving i.p. implants of leukaemia cells preincubated with 35 μg/ml of CPN for 30 min or 60 min gave 80 and 90% survivors respectively. However, when the VCN or CPN treated L 1210 cells were implanted in the peritoneal cavity of mice whose immune status was suppressed by prior cyclophosphamide treatment, there was no evidence of the rejection of implants and all the mice died. Also, in the control groups of normal mice inoculated with $10^3$ or $10^5$ L 1210 cells all the mice died from tumour growth.

*Fate of VCN-treated L* 1210 *cells in the peritoneal cavity of DBA/2 mice*

In order to follow the fate of enzyme treated leukaemia cells in the peritoneal cavity of normal DBA/2 mice, 15 mice were inoculated i.p. with $10^5$ VCN treated L 1210 cells/mouse ($10^5$ cells/50 u VCN/ml for 30 min). After the inoculation peritoneal washings were made at various intervals, by rinsing the peritoneal cavity with 1–2 ml of TCM 199. These suspensions (1 ml) were inoculated into groups of 15–20 normal mice. The washings harvested 4–6 days after the implantations of enzyme treated cells failed to induce

TABLE II.—*Induction of Immunoprotection in DBA/2 Mice against L* 1210 *Leukaemia by Neuraminidase Treated L* 1210 *Cells*

| Mice surviving previous i.p. inoculation of | Total no. of mice | Interval between primary injection and challenge (weeks) | Challenge dose and route $10^3$ i.p. | $10^3$ s.c. | $10^5$ i.p. | $10^5$ s.c. | $10^4$ EAT cells (i.p.) |
|---|---|---|---|---|---|---|---|
| L 1210 cells preincubated with VCN (50 u/ml/$10^5$ cells) for 30 min | 14 | 3 | 3/3* | 3/3 | 3/3 | | |
| | | 6 | 3/3 | | 2/2 | | |
| L 1210 cells preincubated with VCN (50 u/ml/$10^5$ cells) for 60 min | 16 | 3 | 4/4 | 3/3 | 3/3 | | 0/4 |
| | | 6 | 2/2 | | | | |
| L 1210 cells preincubated with VCN (250 u/ml/$10^5$ cells) for 30 min | 20 | 3 | 3/3 | 3/3 | 4/4 | 3/3 | |
| | | 6 | 3/3 | 2/2 | 2/2 | | |
| L 1210 cells preincubated with VCN (250 u/ml/$10^5$ cells) for 60 min | 20 | 3 | 3/5 | 4/5 | 1/5 | 0/5 | |
| L 1210 cells preincubated with CPN (15 $\mu$g/ml/$10^5$ cells) for 30 min | 16 | 3 | 3/3 | 4/4 | 3/3 | 2/2 | |
| | | 6 | 4/4 | | | | |
| L 1210 cells preincubated with CPN (15 $\mu$g/ml/$10^5$ cells) for 60 min | 17 | 3 | 3/3 | | 4/4 | | 0/3 |
| | | 6 | 3/3 | | 4/4 | | |
| L 1210 cells preincubated with CPN (35 $\mu$g/ml/$10^5$ cells) for 30 min | 16 | 3 | 3/3 | 2/2 | 4/4 | | |
| | | 6 | 3/3 | | 2/2 | 2/2 | |
| L 1210 cells preincubated with CPN (35 $\mu$g/ml/$10^5$ cells) for 60 min | 18 | 3 | 2/5 | 3/5 | 0/3 | 1/5 | |
| Mice (normal controls) | 25 | | 0/5 | 0/5 | 0/5 | 0/5 | 0/5 |

* 40 days survivors (No./Total).

tumours whereas the early washings regularly caused the death of the inoculated mice. This suggests that beyond 4–6 days viable tumour cells were absent in the washings, since the presence of very few tumour cells would be capable of producing fatal leukaemia.

*Immunoprotection induced by enzyme treated L* 1210 *cells*

The different groups of mice surviving i.p. implantation of L 1210 cells pretreated with neuraminidase preparations were subsequently challenged with graded doses of L 1210 cells s.c. or i.p. at intervals of either 3 weeks or 6 weeks following the implantation of enzyme treated leukaemia cells (Table II). One group of animals which had survived primary implantation of enzyme treated L 1210 cells was challenged with $10^4$ Ehrlich ascites tumour cells (EAT). Groups of normal mice were inoculated with corresponding inocula i.p. or s.c. and included as controls. Mice which had survived a primary i.p. injection of L 1210 cells preincubated with 50 u/ml of VCN or 15 $\mu$g/ml of CPN for either 30 min or 60 min were completely protected against i.p. or s.c. challenge doses of $10^3$ or $10^5$ cells, whether challenged 3 weeks or 6 weeks after the implantation of enzyme treated leukaemia cells. Mice surviving a primary implantation of L 1210 cells preincubated with 250 u/ml of VCN or 35 $\mu$g/ml of CPN for 30 min also showed complete protection when challenged at intervals of 3 weeks or 6 weeks with either $10^3$ or $10^5$ cells. However, mice surviving an i.p. implantation of L 1210 cells preincubated with 250 u/ml of VCN or 35 $\mu$g/ml of CPN for 60 min showed survival rates of only 60% and 40% respectively when challenged i.p. with $10^3$ cells 3 weeks following the implantation of enzyme treated L 1210 cells. If the challenge of mice pretreated

TABLE III.—*Growth of L 1210 Cells in Normal DBA/2 Mice Pretreated with Serum or Peritoneal Exudate Cells (PE) from Mice which Survived i.p. Implantation of VCN-treated L 1210 Cells*

| Material transferred (i.p.) | Interval between injection of VCN treated cells and collection of serum or (PE) cells* | Ratio of (PE) cells to L 1210 cells | i.p. challenge with $10^4$ L 1210 cells: 80 days survivors | i.p. challenge with $10^4$ L 1210 cells: AST (days) | i.p. challenge with $10^4$ EAT cells 40 days survivors |
|---|---|---|---|---|---|
| 1 ml serum (donors received $10^5$ VCN treated L 1210 cells, i.p.) | 3 weeks | | 8/8 | | 0/6 |
| 1 ml serum (donors received $10^5$ VCN treated L 1210 cells, i.p.) | 2 months | | 0/10 | 10·2 | 0/5 |
| 1 ml serum (donors, normal DBA/2 mice) | | | 0/5 | 11·0 | |
| PE cells (donors received $10^5$ VCN treated L 1210 cells, i.p.) | 3 weeks | 800 : 1 | 15/15 | | 0/5 |
| | | 200 : 1 | 9/10 | 14·0 | |
| | | 100 : 1 | 6/10 | 16·0 | |
| | | 10 : 1 | 0/10 | 10·0 | |
| PE cells (donors received $10^5$ VCN treated L 1210 cells, i.p.) | 2 months | 800 : 1 | 7/12 | 19·0 | |
| | | 200 : 1 | 6/10 | 16·0 | |
| | | 100 : 1 | 4/10 | | |
| | | 10 : 1 | 0/10 | 9·0 | |
| PE cells (donors normal DBA/2 mice) | | 800 : 1 | 2/12 | 10·0 | |
| 1 ml TCM 199 | | | 0/5 | 10·0 | |

* Peritoneal exudate cells (PE) contained about 85–90% macrophages.

with neuraminidase-treated L 1210 cells ($10^5$ cells, 50 u/ml for 30 min) was made i.p. with $10^4$ EAT cells no protection could be observed.

*Fate of the challenge inoculum in the peritoneal cavity of immune mice*

It was of interest to ascertain the fate of a challenge with normal L 1210 cells implanted in the peritoneal cavity of mice surviving the primary inoculum of enzyme treated L 1210 cells. Attempts were therefore made to isolate L 1210 cells from the peritoneal cavity of challenged mice that did not develop tumours 30 days following the challenge. The peritoneal washings of the survivors were injected i.p. (1–2 ml/mouse) into a group of 20 normal mice. None of the treated mice developed tumours. This implies that the immunity has a lethal rather than static effect on L 1210 cells used for challenge.

*Passive transfer of immunity with serum and PE cells*

Twenty mice were given i.p. administration of $10^5$ VCN treated L 1210 cells (50 u/ml/$10^5$ cells for 60 min) per mouse. After intervals of 3 weeks or 2 months following the implantation, groups of 10 mice were used for preparing the serum and PE cells for passive transfer experiments. The results presented in Table III indicate that sera collected 3 weeks following the implantation of enzyme treated L 1210 cells when administered i.p. (1 ml per mouse) 30 min before the challenge with $10^4$ untreated L 1210 cells protected the mice completely. The serum did not protect mice which received i.p. injection of $10^4$ EAT cells. The recipients of sera from normal donors showed 100% lethality. The protection by serum against L 1210 leukaemia was not observed in the samples of serum collected 2 months after the implantation of enzyme treated leukaemia cells.

PE cells collected 3 weeks after the implantation of enzyme treated cells when transferred i.p. 30 min before tumour challenge completely rejected the growth of $10^4$ L 1210 cells (the ratio between leukaemia cells to PE cells being 1 : 800). The administration of PE cells of immune mice and L 1210 cells at ratios of 200 : 1,

100 : 1 and 10 : 1 resulted in 90%, 60% and 0% survivors respectively among the normal recipients. The PE cells collected 2 months after the implantation of enzyme treated cells protected 7 out of a total of 12 mice against a challenge dose of $10^4$ L 1210 cells when the ratio between L 1210 cells and PE cells was 1 : 800. The 5 mice which died of ascites tumour had an AST of 19 days compared with a 10–11 days survival period of controls. When the leukaemia cells and PE cells were administered at ratios of 1 : 200, 1 : 100 and 1 : 10, the survival rates among the recipients were 60, 40 and 0% respectively. The PE cells from normal donors failed to confer a protection in normal DBA/2 mice against $10^4$ L 1210 cells when the ratio between leukaemia cells to PE cells was 1 : 800. The PE cells of immune mice were unable to protect the recipients against a challenge with $10^4$ EAT cells when the ratio of PE cells to EAT cells was 800 : 1.

### DISCUSSION

Bagshawe and Currie (1968) have previously found that L 1210 cells preincubated with VCN lost the property to kill normally susceptible (C 57BL/DBA/2$F_1$) mice following i.p. inoculation, whereas all pre-irradiated mice died from tumour growth. The results of the present study confirm that the *in vitro* incubation of L 1210 cells with either VCN or CPN destroyed their capacity to induce tumours when implanted i.p. in intact isogenic DBA/2 mice. On the other hand, the mice whose immune status was suppressed by prior treatment with the drug cyclophosphamide, permitted the progressive growth of enzyme treated leukaemia cells, resulting eventually in their death.

Neuraminidase-induced alteration in tumour transplantability has been reported in a variety of other tumour host systems (Sanford, 1967; Lindenmann and Klein, 1967; Currie and Bagshawe, 1968, 1969). However, the mechanism(s) which might account for the failure of enzyme treated tumour cells to grow in otherwise susceptible hosts is not yet clear. It has been suggested that neuraminidase treatment leads to an increase in the immunogenicity of the treated cells, resulting in an effective recognition by the host immune system (Bagshawe and Currie, 1968; Currie and Bagshawe, 1969; Sanford, 1967; Bekesi, St Arneault and Holland, 1971). Cormack (1970), however, working with Walker 256 tumour in rats attributed the increased survival rates of rats receiving enzyme treated tumour cells to their reduced attachment to the mesothelial membrane. He ruled out neuraminidase-induced increase in immunogenicity as an explanation for the reduced lethality among the recipients to the neuraminidase treated tumour cells. On the contrary, he concluded that neuraminidase treatment renders the tumour cells less antigenic.

It is obvious from the present results that the *in vitro* incubation of $10^5$ L 1210 cells with VCN at a concentration of 50 u/ml or 15 $\mu$g/ml of CPN for a period of 30 min was sufficient to retard the tumour development significantly in the peritoneal cavities of normal recipients. The fact that following implantation, the enzyme treated tumour cells in immunosuppressive treated mice showed uninhibited growth supports the assumption that the *in vitro* treatment with the enzyme neuraminidase does not influence the viability, nor any regression in the malignant state of the treated tumour cells, and that the disappearance of the treated tumour cells in normal mice is due to immunological host factors. Apparently there exists a lethal effect on the enzyme treated cells in the peritoneal cavity of the normal DBA/2 mice and as a consequence of this the treated tumour cells disappear totally from the peritoneal cavity of the mice within 4–6 days. Recent *in vitro* studies have suggested the existence of heat-labile factor(s) in the normal sera of some, but not all, mouse strains which are capable of exerting a cytotoxic effect on neuraminidase treated but not on untreated tumour cells (Sanford and Condington, 1971; Sethi and Brandis, 1972). Working with lymphoid cells Ray, Gewurz and Simmons (1971) have demonstrated increased cytolysis of VCN treated cells by complement. It would seem unlikely, however, that the *in vivo* destruction of enzyme treated L 1210 cells is brought about by mechanism(s) which involves the participation of the whole complement system since the DBA/2 strain of mice has a complement deficiency (Rosenberg and Tachibana, 1962).

Bagshawe and Currie (1968) reported that mice surviving primary i.p. implant of VCN treated L 1210 cells developed immunity to subsequent i.p. challenge with untreated leukaemia cells. However, the immunity obtained was relatively weak, since the mice could not be protected against a challenge dose of more than $8 \times 10^3$ tumour cells. In the present study we have been able to demonstrate that the recipients of enzyme treated L 1210 cells become refractory not only to i.p. challenge but also to subcutaneous implants of this leukaemia. This suggests that the evoked immunity was not local in the sense that it is confined to the organ which had been previously implanted with enzyme treated cells. The specificity of the induced immune response is indicated by the fact that the mice were unable to reject a low i.p. challenge dose of EAT cells. It is also obvious that mice surviving an i.p. implant of $10^5$ L 1210 cells preincubated with 50 u/ml of VCN or 15 $\mu$g/ml of CPN for either 30 or 60 min proved completely immune to subsequent challenge inocula of $10^3$ or $10^5$ leukaemia cells. However, the immune response induced by L 1210 cells preincubated with 250 u/ml of VCN or 35 $\mu$g/ml of CPN for 60 min failed to arrest the growth of a challenge dose of $10^5$ L 1210 cells although protection was achieved against $10^3$ L 1210 cells. Conceivably the preincubation of L 1210 cells with high neuraminidase concentrations for prolonged periods resulted in the destruction of the immunogenic capacity of the leukaemia cells (Bekesi *et al.*, 1971). The failure of Bagshawe and Currie (1968) to achieve protection of mice which survived a primary injection of VCN treated L 1210 cells against challenge doses higher than $8 \times 10^3$ could be attributable to the reduced immunogenicity of the enzyme treated L 1210 cell preparations as a result of incubation with a high VCN concentration, *i.e.* 500 u/$2 \times 10^6$ cells/ml for 30 min.

Judging from the results of the experiments carried out in an attempt to transfer the immunity from the mice which had received the enzyme treated L 1210 cells to normal DBA/2 mice, it appears that the immune responses evoked by enzyme treated L 1210 cells were both humoral and cellular. Passive transfer of immunity was successfully achieved by i.p. administration of serum obtained from donors which had been implanted with enzyme treated tumour cells 21 days before. The serum collected from donors which had received the enzyme treated L 1210 cells 2 months previously, however, was not effective in preventing the growth of $10^3$ L 1210 cells. Adoptive transfer of immunity was also achieved by PE cells from animals which had received enzyme treated tumour cells. This type of transfer was, however, effective as late as 2 months after the donors had received enzyme treated L 1210 cells. Since the PE cell population used in transfer experiments consisted mainly of macrophages (85–90%), it implies that macrophages were the effector cells in the rejection of the tumour. It may be mentioned, however, that strictly speaking the transfer of immunity by PE cells does not exclude the participation of humoral antibodies in the rejection phenomenon because of the possible presence of antibody forming cells among the transferred population and/or the role of cytophilic antibody(ies) at the surface of the macrophages. Furthermore, in spite of its strain specificity a weak immune response against L 1210 leukaemia has been reported to exist in DBA/2 mice (Mihich, 1969) and as such the co-operation of this host defence response, which is relatively inefficient *per se* in the rejection of L 1210 leukaemia cells observed in our transfer experiments, cannot be completely ruled out. It is not yet known whether a qualitative difference exists between the immune response transferred by the serum and that transferred by PE cells.

Thus, whereas the results of the present study suggest that neuraminidase treatment of tumour cells results in an increase in their immunogenicity, it is also clear that prolonged treatment with higher enzyme concentrations can abolish the immunogenic capacity of the treated preparations. The nature of immunogen(s) involved and the exact mode of origin of highly immunogenic L 1210 cells following neuraminidase treatment is still obscure.

Whatever the nature of the immunogen(s) involved may be, neuraminidase treatment appears to be an effective procedure for producing " attenuated " vaccines for an otherwise susceptible host. The possible use of such " attenuated " tumour vaccines in the immunotherapy of cancer is obvious. In preliminary unpublished experiments we have been able to reduce successfully the incidence of lethality due to advanced L 1210 leukaemia, and have also achieved a significant prolongation in the life span of leukaemic mice by repeated interdermal injections of neuraminidase treated living leukaemia cells and also by neuraminidase treated membrane preparations of this leukaemia cell type.

### REFERENCES

BAGSHAWE, K. D. & CURRIE, G. A. (1968) Immunogenicity of L 1210 Murine Leukaemia Cells after Treatment with Neuraminidase. *Nature, Lond.*, **218**, 1254.

BEKESI, J. G., ST ARNEAULT, G. & HOLLAND, J. F. (1971) Increase of Leukaemia L 1210 Immunogenicity by *Vibrio cholerae* Neuraminidase Treatment. *Cancer Res.*, **31**, 2130.

BERENBAUM, M. C. & BROWN, I. N. (1964) Dose-response Relationships for Agents Inhibiting the Immune Response. *Immunology*, **7**, 65.

CORMACK, D. (1970) Effect of Enzymatic Removal of Cell Surface Sialic Acid on the Adherence of Walker 256 Tumor Cells to Mesothelial Membrane. *Cancer Res.*, **30**, 1459.

CURRIE, G. A. & BAGSHAWE, K. D. (1968) The Role of Sialic Acid in Antigenic Expression: Further Studies of the Landschütz Ascites Tumour. *Br. J. Cancer*, **22**, 843.

CURRIE, G. A. & BAGSHAWE, K. D. (1969) Tumour Specific Immunogenicity of Methylcholanthrene-induced Sarcoma Cells after Incubation in Neuraminidase. *Br. J. Cancer*, **23**, 141.

LAW, L. W., DUNN, T. B., BOYLE, P. J. & MILLER, J. H. (1949) Observations on the Effect of a Folic Acid Antagonist on Transplantable Lymphoid Leukaemias in Mice. *J. natn. Cancer Inst.*, **10**, 179.

LEITER, J., ABBOTT, B. J. & SCHEPARTZ, S. A. (1964) Screening Data from Cancer Chemotherapy National Service Center Screening Laboratories. *Cancer Res.*, **24** (II), 1093.

LINDENMANN, J. & KLEIN, P. A. (1967) Immunologic Aspects of Viral Oncolysis. In *Recent Results in Cancer Research*, vol. 9. New York: Springer-Verlag, p. 66.

MIHICH, E. (1969) Combined Effects of Chemotherapy and Immunity against Leukaemia L 1210 in DBA/2 mice. *Cancer Res.*, **29**, 848.

RAY, P. K., GEWURZ, H. & SIMMONS, R. L. (1971) Complement Sensitivity of Neuraminidase Treated Lymphoid Cells. *Transplantation*, **12**, 327.

ROSENBERG, L. T. & TACHIBANA, D. K. (1962) Activity of Mouse Complement. *J. Immun.*, **89**, 861.

SANFORD, B. H. (1967) An Alteration in Tumor Histocompatibility Induced by Neuraminidase. *Transplantation*, **5**, 1273.

SANFORD, B. H. & CONDINGTON, J. F. (1971) Further Studies on the Effect of Neuraminidase on Tumor Cell Transplantability. *Tissue Antigens*, **1**, 153.

SETHI, K. K. & BRANDIS, H. (1972) *In vitro* Cytotoxicity of Normal Serum Factor(s) on Neuraminidase Treated Ehrlich Ascites Tumor Cells and Murine Leukaemia L 1210 Cells. *Z. ImmunForsch.* **143**, 426.

SIMMONS, R. L., RIOS, A., LUNDGREN, G., RAY, P. K., MCKHANN, C. F. & HAYWOOD, G. R. (1971) Immunospecific Regression of Methylcholanthrene Fibrosarcoma with the Use of Neuraminidase. *Surgery, St Louis*, **70**, 38.

SKIPPER, H. E., SCHABEL, F. M. JR. & WILOX, W. S. (1967) Experimental Evaluation of Potential Anticancer Agents. XXI. Scheduling of Arabinosylcytosine to take Advantage of its S-phase Specificity against Leukaemia Cells. *Cancer Chemother. Rep.*, **51**, 125.

WOODRUFF, J. J. & GESNER, B. M. (1969) The Effect of Neuraminidase on the Fate of Transfused Lymphocytes. *J. exp. Med.*, **129**, 551.